# Selective Splenic Artery Embolization for Refractory Transjugular Intrahepatic Portosystemic Shunt-Induced Hepatic Encephalopathy

**DOI:** 10.7759/cureus.109526

**Published:** 2026-05-23

**Authors:** Luo Zuo

**Affiliations:** 1 Department of Gastroenterology, The Second Affiliated Hospital of Chengdu Medical College, Nuclear Industry 416 Hospital, Chengdu, CHN

**Keywords:** cirrhosis, hepatic encephalopathy, immunity disfunction, selective splenic artery embolization, transjugular intrahepatic portosystemic shun

## Abstract

Transjugular intrahepatic portosystemic shunt (TIPS) is a mainstay treatment for complications of portal hypertension. Despite advances in covered stents, hepatic encephalopathy (HE) remains a common complication, affecting 21%-53% of patients within one year. Refractory HE, often resistant to medical therapy, poses significant management challenges. We present a case of refractory post-TIPS HE successfully managed with selective splenic artery embolization (SSAE), underscoring its therapeutic potential.

## Introduction

Transjugular intrahepatic portosystemic shunt (TIPS) is an established treatment for portal hypertension-related complications, including variceal bleeding and refractory ascites, as recommended by current American Association for the Study of Liver Diseases (AASLD) guidance [1,2]. Although advances in covered stent technology have improved shunt patency, post-TIPS hepatic encephalopathy (HE) remains a common complication, affecting 21%-53% of patients within one year [3].

Refractory HE represents a major clinical challenge, particularly in patients who fail optimal medical therapy [4]. Shunt reduction or occlusion may alleviate HE but carries the risk of worsening portal hypertension and recurrent variceal hemorrhage, especially in patients with elevated pre-TIPS portosystemic pressure gradients (PPG >20 mmHg) [5].

Selective splenic artery embolization (SSAE) has emerged as a potential alternative approach by decreasing splenic venous inflow and improving hepatic arterial perfusion without compromising portal decompression. Herein, we present a case of refractory post-TIPS HE successfully treated with SSAE, highlighting an alternative hemodynamic strategy that may control encephalopathy while preserving portal decompression. This case emphasizes important clinical decision-making when conventional shunt reduction carries substantial risk.

## Case presentation

**Figure 1:**
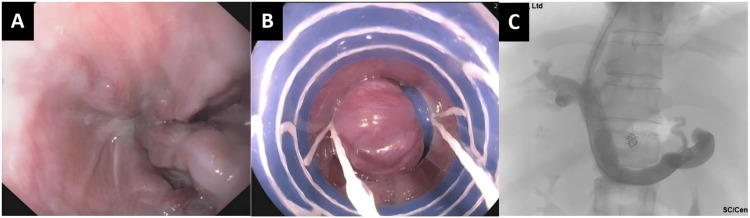
Treatment of esophageal variceal bleeding before selective splenic artery embolization. A. Upper gastrointestinal endoscopy revealed large, tumor-like esophageal varices. B. The esophageal varices were treated with band ligation. C. Portal venogram obtained during transjugular intrahepatic portosystemic shunt insertion.

A 45-year-old man with hepatitis B-related cirrhosis presented with recurrent hematemesis and melena. On admission, the hemoglobin level was 7.8 g/dL, and the patient was hemodynamically unstable. The patient had a prior history of variceal hemorrhage. Following the bleeding episode, the patient underwent endoscopic variceal ligation (Figures 1A-1B) and received secondary prophylaxis with non-selective β-blockers.

Laboratory evaluation demonstrated total bilirubin of 3.79 mg/dL, albumin of 3.0 g/dL, creatinine of 0.76 mg/dL, INR of 1.28, serum ammonia of 53.4 μmol/L, and a Model for End-Stage Liver Disease (MELD) score of 14. Hypersplenism was evident, with leukopenia (WBC 2.88 × 10³/μL) and thrombocytopenia (platelets 55 × 10³/μL).

The patient developed refractory variceal bleeding requiring emergency Minnesota tube placement. Salvage TIPS was subsequently performed. The PPG decreased from 36 mmHg pre-procedure to 15 mmHg post-procedure (Figure 1C).

Post-TIPS complications and intervention

Twenty-five days after TIPS placement, the patient developed Grade II HE, presenting with lethargy and disorientation without impaired consciousness, precipitated by constipation. The symptoms were temporarily relieved with lactulose and rifaximin therapy. However, over the subsequent two years, the patient experienced recurrent episodes of Grade I-II HE, primarily triggered by constipation and bacterial infections.

Pre-procedural evaluation prior to SSAE demonstrated total bilirubin of 4.3 mg/dL, albumin of 2.68 g/dL, creatinine of 0.77 mg/dL, INR of 1.90, ammonia of 298 μmol/L, and a MELD score of 19. Hematologic parameters showed anemia (RBC 3.6 × 10⁶/μL), leukopenia (WBC 1.86 × 10³/μL), and thrombocytopenia (platelets 33 × 10³/μL).

SSAE procedure

**Figure 2:**
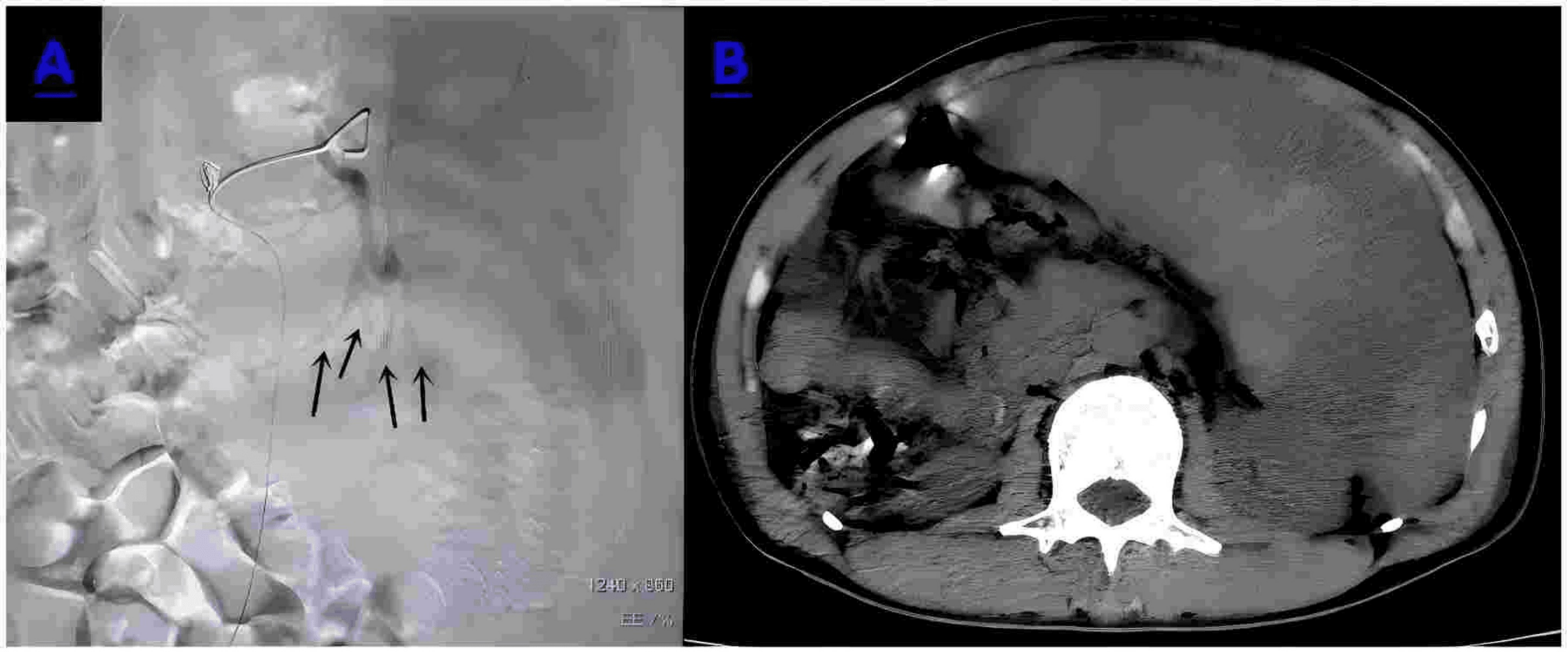
Selective splenic artery embolization procedure. A. Fluoroscopic image demonstrating occlusion of the inferior branch of the splenic artery following embolization. B. Contrast-enhanced CT image showing low attenuation of the inferior splenic pole, consistent with splenic infarction.

Under fluoroscopic guidance, 1-mm gelatin sponge particles were injected into a distal splenic arterial branch, achieving 40% splenic volume embolization (Figure 2).

Clinical outcomes

Within 48 hours post-SSAE, the patient exhibited complete resolution of encephalopathic symptoms, with alert mental status, orientation to time, place, and person, and no asterixis. Serial laboratory monitoring demonstrated progressive improvement in total bilirubin (4.3→1.8 mg/dL), albumin (2.68→3.29 g/dL), INR (1.90→1.49), and ammonia (298→55.4 μmol/L) (Table 1). At 18-month follow-up, there was no HE recurrence or variceal rebleeding.

**Table 1:** Laboratory values before and after selective splenic artery embolization. SSAE: Selective splenic artery embolization; RR: Reference range.

Time point	Total bilirubin, mg/dL (RR: 0.13-1.17)	Albumin, g/dL (RR: 3.5-5.5)	Platelet count, × 10³/μL (RR: 10.0-40.0)	Leukocyte count, × 10³/μL (RR: 3.5-9.5)	Ammonia, μmol/L (RR: 18-72)	INR (RR: 0.8-1.2)
Pre-SSAE	4.3	2.68	33	1.86	298	1.9
7 days post-SSAE	3.95	2.72	37	2.65	55.1	1.74
14 days post-SSAE	2.56	2.88	87	2.45	31.5	1.58
1 month post-SSAE	1.73	2.32	61	2.08	30.7	1.69
3 months post-SSAE	2.47	2.93	62	3.1	55.4	1.48
6 months post-SSAE	3.03	2.8	65	3.4	58.8	1.6
12 months post-SSAE	3.98	2.81	62	3.6	66.7	1.68
18 months post-SSAE	2.02	3.29	56	3.96	55.4	1.49

## Discussion

Although shunt reduction is a potential therapeutic option for refractory post-TIPS HE, it may compromise portal decompression and increase the risk of recurrent portal hypertension-related complications, including variceal rebleeding, particularly in patients with a pre-TIPS PPG exceeding 20 mmHg [5]. In the present case, the markedly elevated baseline PPG of 36 mmHg rendered shunt modification unfavorable, thereby necessitating consideration of alternative therapeutic strategies.

TIPS diverts portal venous flow, reducing first-pass hepatic clearance of ammonia and other neurotoxins. This may activate the hepatic arterial buffer response (HABR) to help maintain hepatic perfusion [6]. However, inadequate HABR compensation may exacerbate ammonia accumulation and neurotoxin-mediated neuronal injury. Selective splenic artery embolization (SSAE) may augment HABR by reducing splenic venous return, thereby increasing hepatic arterial flow and potentially enhancing ammonia clearance, a mechanism supported by Ishikawa et al. Post-SSAE improvements in serum albumin and total bilirubin further support this hypothesis.

Cirrhosis-induced splenomegaly drives hypersplenism and immune dysfunction, characterized in this case by leukopenia (1.86 × 10³/μL) and thrombocytopenia (33 × 10³/μL). SSAE mitigates these effects by reducing splenic volume, with 40% embolization achieved in this case, which was associated with improved leukocyte and platelet counts post-SSAE (WBC increased to 3.96 × 10³/μL; platelets increased to 56 × 10³/μL). This may help reduce infection risk, which is clinically relevant in this patient with recurrent infection-triggered HE episodes.

This case underscores SSAE’s potential dual therapeutic role: (1) balancing portosystemic shunting while preserving hepatic perfusion and (2) improving hypersplenism-related cytopenias and immune dysfunction [7]. Notably, the patient achieved complete HE resolution within 48 hours post-SSAE, with sustained remission at follow-up. These outcomes align with studies reporting improvement in HE after splenic artery embolization.

## Conclusions

This case highlights SSAE as a potential alternative for refractory post-TIPS HE, particularly in patients with contraindications to shunt reduction. By integrating hemodynamic optimization and potential immune reconstitution, SSAE may offer a multimodal therapeutic strategy for this complex clinical scenario.
